# Wild Rodents and Novel Human Pathogen *Candidatus* Neoehrlichia mikurensis, Southern Sweden

**DOI:** 10.3201/eid1709.101058

**Published:** 2011-09

**Authors:** Martin Andersson, Lars Råberg

**Affiliations:** Author affiliation: Lund University, Lund, Sweden

**Keywords:** Anaplasmataceae, Candidatus Neoehrlichia mikurensis, Anaplasma phagocytophilum, Bartonella, bacteria, tick-borne disease, zoonotic disease, wild rodents, Rickettsia, Sweden, dispatch

## Abstract

We examined small mammals as hosts for *Anaplasmataceae* in southern Sweden. Of 771 rodents, 68 (8.8%) were infected by *Candidatus* Neoehrlichia mikurensis, but no other *Anaplasmataceae* were found. *Candidatus* N. mikurensis has recently been found in human patients in Germany, Switzerland, and Sweden, which suggests that this could be an emerging pathogen in Europe.

The rickettsial family *Anaplasmataceae* contains several tick-transmitted bacteria of considerable medical and veterinary importance, including known human pathogens such as *Anaplasma phagocytophilum* and *Ehrlichia chaffeensis*. Recently, a case of human disease in a Swedish patient with recurrent fever episodes caused by another member of *Anaplasmataceae* was reported. The infectious agent was shown by PCR amplification of the 16S rRNA gene to be identical to *Candidatus* Neoehrlichia mikurensis (*1*).

This published report was followed by the description of a patient from Switzerland (*2*) and 2 German patients with severe febrile illness caused by *Candidatus* N. mikurensis (*3*). This member of the family *Anaplasmataceae* was first discovered in ticks in the Netherlands in 1999; it was originally designated as an *Ehrlichia* spp.–like species (*4*). Similar organisms were later detected in Russia (*5*) as well as in other parts of Europe (*6*). In 2004, a closely related organism was detected in Japan in wild rodents and was named *Candidatus* N. mikurensis (*7*). In the United States, a similar organism (*Candidatus* N. lotori) has been detected in wild raccoons (*8*). The distribution and reservoir hosts of *Candidatus* N. mikurensis in Europe are largely unknown. It has, however, been detected in a blood sample from a single bank vole in Italy (*9*), indicating that rodents are possible reservoir hosts.

In this study, we collected blood samples from 7 small mammal species (rodents and shrews) at 5 localities in southern Sweden to investigate their role as hosts for *Anaplasmataceae* to determine whether these small mammals serve as natural hosts for *Candidatus* N. mikurensis.

## The Study

**Figure 1:**
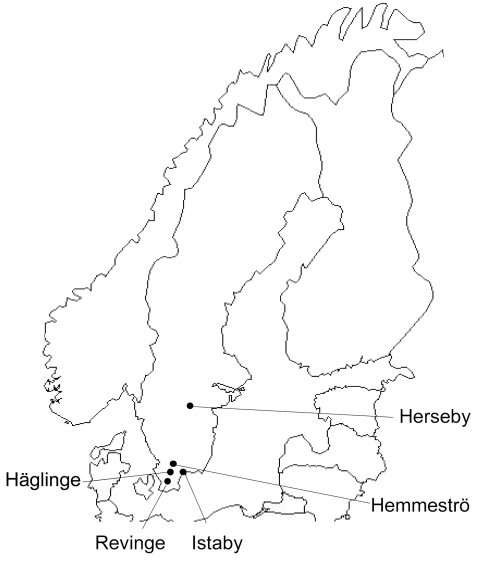
Collection locations for rodents and shrews tested for *Candidatus* Neoehrlichia mikurensis and *Bartonella* spp. infections, southern Sweden, 2008. Prevalence of infection: Häglinge, n = 45 infections, 0% *Candidatus* N. mikurensis, 44.4% *Bartonella* spp.; Revinge, n = 623 infections, 9.3% *Candidatus* N. mikurensis, 33.7% *Bartonella* spp.; Istaby, n = 53 infections, 3.8% *Candidatus* N. mikurensis, 34% *Bartonella* spp.; Hemmeströ, n = 64 infections, 4.7% *Candidatus* N. mikurensis, 39.1% *Bartonella* spp.; Herseby, n = 49 infections, 12.5% *Candidatus* N. mikurensis, 45.0% *Bartonella* spp.

From May through October 2008, we trapped small mammals at 5 sites in southern Sweden (Figure 1). Animals were captured with live traps and released after sampling. Our primary objective was to obtain samples from bank voles, *Myodes glareolus,* but other species were also sampled when captured. A total of 829 animals of 7 species were caught (Table). Approximately 20 µL of blood was taken from each animal. A nested PCR was performed with primers for *Anaplasmataceae* specific for the 16S rRNA gene (*10*).

**Table:** Species tested and number of animals infected with *Candidatus* Neoehrlichia mikurensis or *Bartonella* spp., Sweden, 2008

Species	No. sampled	*Candidatus* N. mikurensis	*Bartonella* sp.
Bank vole (*Myodes glareolus*)	705	64	232
Field vole *(Microtus agrestis*)	24	2	11
Wood mouse (*Apodemus sylvaticus*)	10	1	4
Yellow-necked mouse (*A. flavicollis*)	25	1	14
*Apodemus* sp.	7	0	5
Common shrew (*Sorex araneus*)	43	0	28
Pygmy shrew (*S. minutus*)	12	0	0
Water shrew (*Neomys fodiens*)	3	0	0
Total	829	68	294

All amplified fragments were sequenced, and a BLAST (http://blast.ncbi.nlm.nih.gov/Blast.cgi) search showed that 68 animals were infected by *Candidatus* N. mikurensis. The primers were chosen to be specific for bacteria belonging to the families *Rickettsiaceae* and *Anaplasmataceae* (*10*), but they also amplified *Bartonella* spp. under the given PCR conditions. In total, 35.5% of the animals were infected by *Bartonella* spp. Double infections with both *Candidatus* N. mikurensis and *Bartonella* spp., as indicated by double peaks on the sequencing chromatogram, occurred in 12 cases.

**Figure 2:**
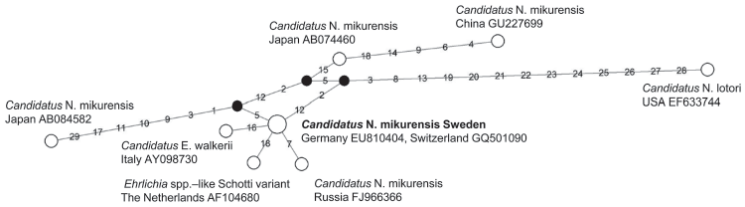
Phylogenetic network of 16s rRNA sequences (1,231 bp) from *Candidatus* Neoehrlichia mikurensis, southern Sweden, 2008. Black nodes indicate intermediate inferred sequences on the most parsimonious route between observed sequences. Numbers on branches represents mutations, numbered according to nucleotide position in the alignment. The sequence obtained in this study is shown in **boldface** and is identical with sequences from human patients in Germany (*3*) and Switzerland (*2*). The Japanese reference strain TK4456 (*7*) showed 99.2% similarity with our sequence, and the North American *Candidatus* N. lotori strain (*12*) showed 98.3% similarity.

To further characterize the obtained *Candidatus* N. mikurensis, we sequenced 1,426 bp of the 16S rRNA and 1,233 bp of the *groEL* gene (*1*). The obtained 16S rRNA *Candidatus* N. mikurensis sequences in this study were identical (1,426/1,426 bp) to sequences obtained from human patients in Germany and Switzerland (*2**,**3*). The *groEL* sequence was identical to the isolate from Germany (1,233/1,233 bp) but differed slightly from the isolate from Switzerland (1,072/1,084 bp, 98.9% pairwise identity). A phylogenetic network containing unique 16s rRNA sequences from *Candidatus* N. mikurensis available at the National Center for Biotechnology Information was made with the program Network 4.5.1.6 (www.fluxus-engineering.com) by using the median-joining algorithm (*11*) (Figure 2).

The prevalence of *Candidatus* N. mikurensis at the 5 sites ranged from 0% to 12.5%, and *Bartonella* spp. occurred in 33.7% to 45.0% of the animals (Figure 1). The prevalence of *Candidatus* N. mikurensis and *Bartonella* spp. in each host species is given in the Table. *Candidatus* N. mikurensis occurred in all 4 rodent species, but not in the shrews; the difference in prevalence between rodents and shrews was statistically significant (p = 0.011, by Fisher exact test). *Bartonella* spp. were found in all rodent species and the common shrew.

## Conclusions

In the present field survey of *Anaplasmataceae* in Swedish rodents, we have amplified 16s rRNA and *groEL* sequences identified as *Candidatus* N. mikurensis identical to sequences obtained from humans. This organism has been amplified from humans with febrile illness on ≥4 occasions (*1**–**3*), demonstrating that *Candidatus* N. mikurensis can cause human infections, at least occasionally.

Apart from the first human case, to our knowledge, *Candidatus* N. mikurensis has not been detected in Sweden. However, we identified *Candidatus* N. mikurensis at 4 of 5 sites, indicating that this organism is widespread in southern Sweden. Identical or similar sequences have been detected in *Ixodes ricinus* ticks from several European countries (*3**–**6**,**13*), showing that it is distributed over a large area of Europe. In contrast, we found no evidence of infection by any other *Anaplasmataceae*. *I. ricinus* is the dominating tick species in Sweden, and animals at all our study sites were heavily infested with larvae of this species and occasionally with nymphs (M. Andersson and L. Råberg, unpub. data). Reservoir hosts are essential for several tick-borne pathogens that lack the capacity for transovarial transmission from female ticks to larvae, such as *A. phagocytophilum* (*14*). Approximately 70% of the tick larvae in Sweden engorge on small mammals, such as voles, mice, and shrews (*15*).

The prevalence of *Candidatus* N. mikurensis was similar in all investigated rodent species in our study with a mean value of 8.8%. These results are consistent with several studies in Japan, which also found *Candidatus* N. mikurensis in several different rodent species (*7**,**10*). This finding indicates that rodents play a role in the natural cycle of *Candidatus* N. mikurensis in Europe and that rodents are likely to be competent reservoir hosts. In contrast, the closely related *Candidatus* N. lotori, found in the United States, seems to use raccoons rather than rodents as hosts (*12*). Whether the European variant of *Candidatus* Neoehrlichia is capable of infecting animals other than rodents and humans remains to be investigated. We found the organism was completely absent in shrews, which suggests that they might not be competent hosts for these bacteria.

We conclude that *Candidatus* N. mikurensis is geographically widespread in southern Sweden and that several rodent species, the main source of blood meals for larvae of *I. ricinus* in Sweden (*15*), can be infected. The relatively high prevalence in common rodent species suggests that the risk for humans and domestic animals to encounter infected *I. ricinus* nymphs and adults is substantial. Thus, *Candidatus* N. mikurensis should be considered when diagnosing bacterial infections associated with tick bites.

## References

[1] Welinder-Olsson C, Kjellin E, Vaht K, Jacobsson S, Wennerås C. First case of human “*Candidatus* Neoehrlichia mikurensis” infection in a febrile patient with chronic lymphocytic leukemia. J Clin Microbiol. 2010;48:1956–9. 10.1128/JCM.02423-09 20220155 PMC2863919

[2] Fehr JS, Bloemberg GV, Ritter C, Hombach M, Lüscher TF, Weber R, Septicemia caused by tick-borne bacterial pathogen *Candidatus* Neoehrlichia mikurensis. Emerg Infect Dis. 2010;16:1127–9. 10.3201/eid1607.091907 20587186 PMC3358111

[3] von Loewenich FD, Geißdörfer W, Disqué C, Matten J, Schett G, Sakka SG, Detection of “*Candidatus* Neoehrlichia mikurensis” in two patients with severe febrile illnesses: evidence for a European sequence variant. J Clin Microbiol. 2010;48:2630–5. 10.1128/JCM.00588-10 20519481 PMC2897504

[4] Schouls LM, Van de Pol I, Rijpkema SG, Schot CS. Detection and identification of *Ehrlichia, Borrelia burgdorferi* sensu lato, and *Bartonella* species in Dutch *Ixodes ricinus* ticks. J Clin Microbiol. 1999;37:2215–22. 10364588 10.1128/jcm.37.7.2215-2222.1999 PMC85121

[5] Alekseev AN, Dubinina HV, Van de Pol I, Schouls LM. Identification of *Ehrlichia* spp, and *Borrelia burgdorferi* in *Ixodes* ticks in the Baltic regions of Russia. J Clin Microbiol. 2001;39:2237–42. 10.1128/JCM.39.6.2237-2242.2001 11376063 PMC88117

[6] Brouqui P, Sanogo YO, Caruso G, Merola F, Raoult D. *Candidatus* Ehrlichia walkerii—a new *Ehrlichia* detected in *Ixodes ricinus* tick collected from asymptomatic humans in northern Italy. Ann N Y Acad Sci. 2003;990:134–40. 10.1111/j.1749-6632.2003.tb07352.x 12860615

[7] Kawahara M, Rikihisa Y, Isogai E, Takahashi M, Misumi H, Suto C, Ultrastructure and phylogenetic analysis of “*Candidatus* Neoehrlichia mikurensis” in the family *Anaplasmataceae*, isolated from wild rats and found in *Ixodes ovatus* ticks. Int J Syst Evol Microbiol. 2004;54:1837–43. 10.1099/ijs.0.63260-0 15388752

[8] Dugan VG, Gaydos JK, Stallknecht DE, Little SE, Beall AD, Mead DG, Detection of *Ehrlichia* spp. in raccoons (*Procyon lotor*) from Georgia. Vector Borne Zoonotic Dis. 2005;5:162–71. 10.1089/vbz.2005.5.162 16011433

[9] Beninati T, Piccolo G, Rizzoli A, Genchi C, Bandi C. *Anaplasmataceae* in wild rodents and roe deer from Trento province (northern Italy). Eur J Clin Microbiol Infect Dis. 2006;25:677–8. 10.1007/s10096-006-0196-x 17047904

[10] Tabara K, Arai S, Kawabuchi T, Itagaki A, Ishihara C, Satoh H, Molecular survey of *Babesia microti, Ehrlichia* species and *Candidatus* Neoehrlichia mikurensis in wild rodents from Shimane Prefecture, Japan. Microbiol Immunol. 2007;51:359–67. 17446675 10.1111/j.1348-0421.2007.tb03923.x

[11] Bandelt H-J, Forster P, Röhl A. Median-joining networks for inferring intraspecific phylogenies. Mol Biol Evol. 1999;16:37–48. 10331250 10.1093/oxfordjournals.molbev.a026036

[12] Yabsley MJ, Murphy SM, Luttrell MP, Wilcox BR, Ruckdeschel C. Raccoons (*Procyon lotor*), but not rodents, are natural and experimental hosts for an ehrlichial organism related to “*Candidatus* Neoehrlichia mikurensis.”. Vet Microbiol. 2008;131:301–8. 10.1016/j.vetmic.2008.04.004 18524503

[13] Spitalská E, Boldis V, Kostanova Z, Kocianova E, Stefanidesova K. Incidence of various tick-borne microorganisms in rodents and ticks of Central Slovakia. Acta Virol. 2008;52:175–9. 18999892

[14] Dumler JS, Barbet AF, Bekker CP, Dasch GA, Palmer GH, Ray SC, Reorganization of genera in the families *Rickettsiaceae* and *Anaplasmataceae* in the order *Rickettsiales*: unification of some species of *Ehrlichia* with *Anaplasma, Cowdria* with *Ehrlichia* and *Ehrlichia* with *Neorickettsia*, descriptions of six new species combinations and designation of *Ehrlichia equi* and “HGE agent” as subjective synonyms of *Ehrlichia phagocytophila.* Int J Syst Evol Microbiol. 2001;51:2145–65. 10.1099/00207713-51-6-2145 11760958

[15] Tälleklint L, Jaenson TG. Transmission of *Borrelia burgdorferi* s.l from mammal reservoirs to the primary vector of Lyme borreliosis, *Ixodes ricinus* (Acari: ixodidae), in Sweden. J Med Entomol. 1994;31:880–6. 7815401 10.1093/jmedent/31.6.880

